# Tissue distribution and temporal and spatial assessment of per- and polyfluoroalkyl substances (PFAS) in smallmouth bass (*Micropterus dolomieu*) in the mid-Atlantic United States

**DOI:** 10.1007/s11356-024-35097-6

**Published:** 2024-09-30

**Authors:** Vicki S. Blazer, Heather L. Walsh, Cheyenne R. Smith, Stephanie E. Gordon, Brandon J. Keplinger, Timothy A. Wertz

**Affiliations:** 1 U.S. Geological Survey, Eastern Ecological Science Center – Leetown Research Laboratory, Kearneysville, WV 25430 USA; 2 West Virginia Division of Natural Resources, Romney, WV 26757 USA; 3 Pennsylvania Department of Environmental Protection, Harrisburg, PA 17101 USA

**Keywords:** Smallmouth bass, PFAS, Plasma, Land use, Tissue distribution, Temporal changes

## Abstract

Per- and polyfluoroalkyl substances (PFAS) have become an environmental issue worldwide. A first step to assessing potential adverse effects on fish populations is to determine if concentrations of concern are present in a region and if so, in which watersheds. Hence, plasma from adult smallmouth bass *Micropterus dolomieu* collected at 10 sites within 4 river systems in the mid-Atlantic region of the United States, from 2014 to 2019, was analyzed for 13 PFAS. These analyses were directed at better understanding the presence and associations with land use attributes in an important sportfish. Four substances, PFOS, PFDA, PFUnA, and PFDoA, were detected in every plasma sample, with PFOS having the highest concentrations. Sites with mean plasma concentrations of PFOS below 100 ng/ml had the lowest percentage of developed landcover in the upstream catchments. Sites with moderate plasma concentrations (mean PFOS concentrations between 220 and 240 ng/ml) had low (< 7.0) percentages of developed land use but high (> 30) percentages of agricultural land use. Sites with mean plasma concentrations of PFOS > 350 ng/ml had the highest percentage of developed land use and the highest number PFAS facilities that included military installations and airports. Four of the sites were part of a long-term monitoring project, and PFAS concentrations of samples collected in spring 2017, 2018, and 2019 were compared. Significant annual differences in plasma concentrations were noted that may relate to sources and climatic factors. Samples were also collected at two sites for tissue (plasma, whole blood, liver, gonad, muscle) distribution analyses with an expanded analyte list of 28 PFAS. Relative tissue distributions were not consistent even within one species of similar ages. Although the long-chained legacy PFAS were generally detected more frequently and at higher concentrations, emerging compounds such as 6:2 FTS and GEN X were detected in a variety of tissues.

## Introduction

Perfluorinated compounds, used in many products including nonstick cookware, cosmetics, carpet and clothing treatments, and aqueous film forming foams (AFFF), have attracted the attention of the public as well as public health and resource management agencies. Called “forever chemicals,” these per- and polyfluoroalkyl substances (PFAS) do not break down easily and can build up in the environment, as well as in tissues of humans and other organisms (Abunada et al. 2020; Ankley et al. 2021; Miranda et al. 2022). Potential links to kidney and testicular cancer, thyroid disease, liver damage, developmental toxicity, ulcerative colitis, high cholesterol, and immune dysfunction in humans have been reported (Sunderland et al. 2019; Fenton et al. 2021). Much less is known about the effects of PFAS on fish populations at environmentally relevant exposure concentrations and in concert with complex contaminant mixtures and other stressors, to which wild aquatic organisms are exposed (Lee et al. 2020; Sinclair et al. 2020; Nayak et al. 2023).

In 2013, the U.S. Geological Survey, in cooperation with state natural resource agencies in Maryland, Pennsylvania, and West Virginia, began monitoring smallmouth bass (SMB, *Micropterus dolomieu*) in response to fish mortalities, numerous types of skin lesions, intersex, and other signs of endocrine disruption and population declines (Blazer et al. 2007, 2010, 2020; Smith et al. 2015; Walsh et al. 2018, 2022; Keplinger et al. 2022). A suite of biological indicators and monthly surface water samples for analyses of pesticides, hormones, phytoestrogens, and pharmaceuticals were monitored at four sites (two in the Potomac River, Maryland, and West Virginia and two in the Susquehanna River, Pennsylvania). PFAS were not part of the analyte list; however, aliquots of SMB plasma from these long-term monitoring sites were archived. In a preliminary study, SMB plasma samples from 2018 were analyzed for 13 PFAS compounds (Blazer et al. 2021) to determine if measurable concentrations of potential concern would be detected. Four compounds (PFOS, PFDA, PFUnA, PFDoA; defined in Table 1) were found in every sample, with PFOS (perfluorooctanesulfonic acid) having the highest concentrations, ranging from 20 to 574 ng/ml. These findings indicated that SMB are exposed to and may be bioaccumulating or bioconcentrating certain PFAS and warranted further investigation. One objective of our study was to evaluate if major temporal changes occurred at these four sites, utilizing archived plasma samples collected in 2017, 2018, and 2019.

**Table 1:** Perfluoroalkyl substances analyzed in smallmouth bass tissues in 2021. Compounds shaded are the thirteen compounds measured in plasma 2014–2019

Chemical name	Abbreviation	Carbon chain length
**Perfluoroalkyl carboxylates**		
Perfluorobutanoic acid	*PFBA*	4
Perfluoro-n-pentanoic acid	*PFPeA*	5
Perfluorohexanoic acid	*PFHxA*	6
Perfluoroheptanoic acid	*PFHpA*	7
Perfluorooctanoic acid	*PFOA*	8
Perfluorononanoic acid	*PFNA*	9
Perfluorodecanoic acid	*PFDA*	10
Perfluoroundecanoic acid	*PFUnA*	11
Perfluorododecanoic acid	*PFDoA*	12
Perfluorotridecanoic acid	PFTrDA	12
Perfluorotetradecanoic acid	PFTeDA	13
**Perfluoroalkyl sulfonates**		
Perfluorobutane sulfonic acid	*PFBS*	4
Perfluoropentane sulfonic acid	PFPeS	5
Perfluorohexane sulfonic acid	*PFHxS*	6
Perfluoroheptane sulfonic acid	PFHpS	7
Perfluorooctane sulfonic acid	*PFOS*	8
Perfluorononane sulfonic acid	PFNS	9
Perfluorodecane sulfonic acid	PFDS	10
**Sulfonamides**		
Perfluorooctane sulfonamide	*PFOSA*	8
**Sulfonamide acetic acids**		
N-Methylperfluorooctanesulfonamidoacetic acid	N-MeFOSAA	8
N-Ethylperfluorooctane sulfonamidoacetic acid	N-EtFOSAA	8
**Fluorotelomer sulfonates**		
4:2 Fluorotelomersulfonic acid	4:2 FTS	4
6:2 Fluorotelomersulfonic acid	6:2 FTS	6
8:2 Fluorotelomersulfonic acid	8:2 FTS	8
**Other**		
4-Dioxa-3H-perfluorononanoic acid	ANONA	6
9-Chlorohexadecafluoro-3-oxaundecane-1-sulfonic acid	9C1-PF3ONS	8
11-Chloroeicosafluoro-3-oxaundecane-1-sulfonic acid	11C1-PF3OYUds	10
HFDO-DA	Gen X	6

The second and third objectives were to better document the spatial variability of PFAS in plasma of SMB and investigate land use attributes that may contribute to exposure. Although land use attributes were considered in a study on PFAS in surface water in Pennsylvania, USA (Breitmeyer et al. 2023), we are not aware of other studies in this region that have assessed land cover and land use attributes such as agricultural practices and known/suspected PFAS sources to better understand concentrations in fish populations.

There has recently been increased monitoring of surface water in the Chesapeake Bay watershed and state agencies measure PFAS in fish muscle/fillets with regard to human health concerns. Measurement of PFAS in fish muscle (or whole body) for human consumption is used to determine human exposure and risk; however, studies in many species including SMB (Shi et al. 2012; Goeritz et al. 2013; Houde et al. 2013; Kaboré et al. 2022; Nilsen et al. 2024) have found muscle concentrations are generally lower than other tissues such as blood and liver. Consequently, muscle concentrations may have less relevance to fish health and population stability. Soerensen et al. (2023) suggested that tissue conversion factors may be estimated from analyses of one tissue but identified a need for species-specific conversion factors. Blood has been suggested as a non-lethal sampling tool for aquatic toxicology (Rodriguez-Jorquera et al. 2018; George et al. 2023), and both blood and liver have been shown to be the primary compartments for PFAS accumulation in numerous fish species (Ankley et al. 2009; Martin et al. 2004; Hoa et al. 2022; Nilsen et al. 2024). Our fourth objective was to analyze additional tissues (whole blood, liver, gonad, muscle) to compare organ distribution and determine if blood could be a nonlethal tissue for monitoring. Additional tissues were collected at the two sites with the highest plasma concentrations and analyzed for an expanded analyte list of 28 PFAS to include both legacy and emerging compounds.

## Methods and materials

### Fish collections

**Fig. 1:**
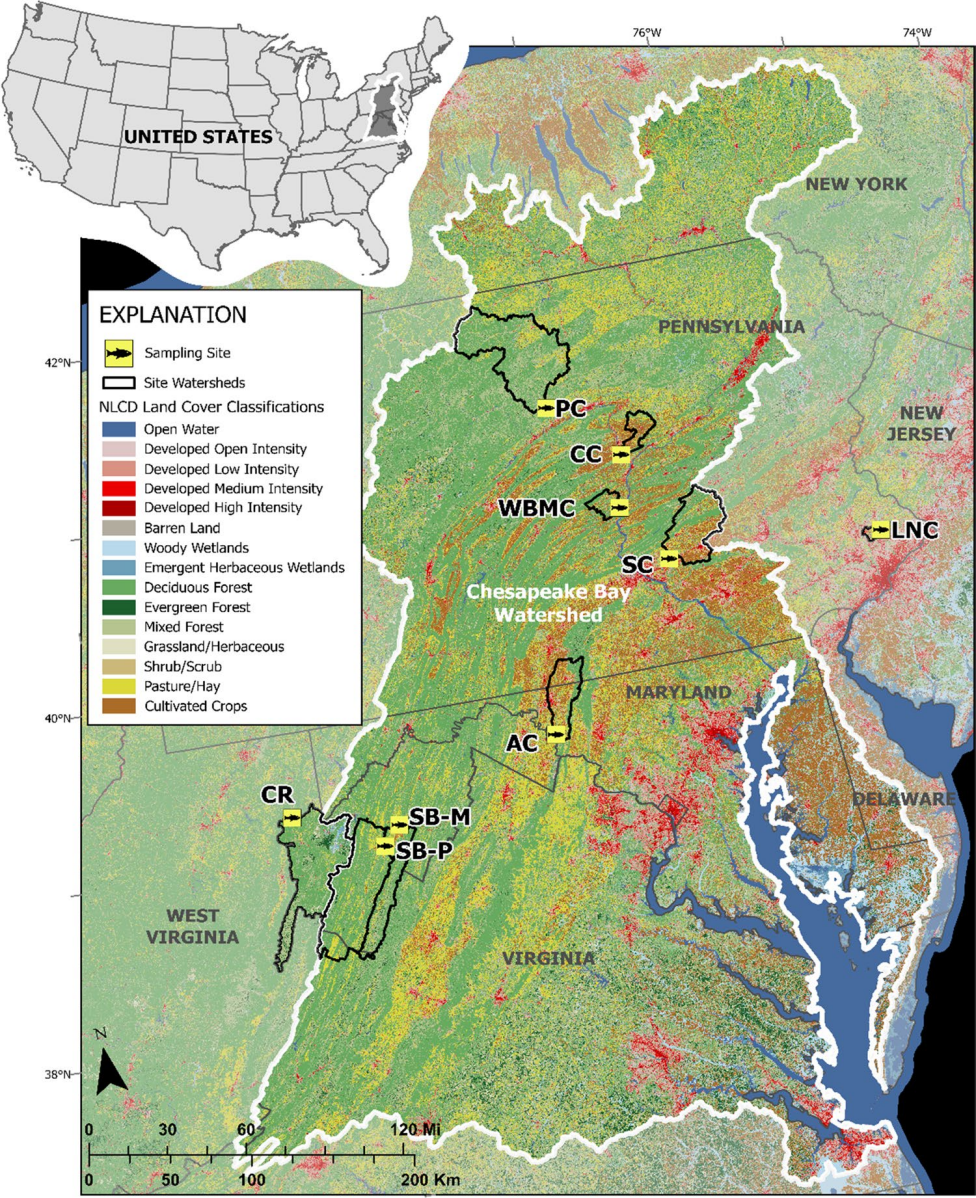
Locations and land cover surrounding smallmouth bass collection sites. Pine Creek (PC), Chillisquaque Creek (CC), West Branch Mahantango Creek (WBMC), Swatara Creek (SC), and Little Neshaminy Creek (LNC) in Pennsylvania; Cheat River (CR), South Branch Potomac River near Moorefield (SB-M), and near Petersburg (SB-P) in West Virginia; Antietam Creek (AC) in the Potomac River in Maryland; Chesapeake Bay watershed outlined in white. Credits: Homer et al. 2020; USCB, 2018, EPA; USGS, 2012; Projection USA Contiguous Albers Equal Area Conic USGS

Archived plasma samples from ongoing monitoring and assessment studies were used for temporal (4 sites) and spatial (10 sites) comparisons of PFAS in SMB. A total of 380 adult SMB were collected by boat electrofishing from multiple rivers (Fig. 1). These included five sites within the Susquehanna River drainage in Pennsylvania. Pine Creek (PC), the northern-most site, and Chillisquaque Creek (CC) are tributaries of the West Branch Susquehanna River and West Branch Mahantango Creek (WBMC) is a tributary of the mainstem Susquehanna River. Sampling for these sites occurred close to the mouth within each creek. One mainstem site (SS) was sampled near Selinsgrove and Swatara Creek (SC); a tributary of the Susquehanna River was sampled near Hershey. Within the Potomac River drainage, fish were collected at two sites in the South Branch Potomac River in West Virginia. One site (SB-M) near Moorefield and one (SB-P) near Petersburg. A site from the mainstem Potomac River at the mouth of Antietam Creek (AC) was collected in Maryland. Sites outside of the Chesapeake Bay watershed included the Cheat River (CR) near Hannahsville, West Virginia, in the Monongahela River watershed and Little Neshaminy Creek (LNC) near Hartsville, Pennsylvania, a tributary of Neshaminy Creek, within the Delaware River drainage (Fig. 1). The four sites for temporal comparisons, PC, WBMC, AC, and SB-M were sampled in spring 2017, 2018 and 2019. Plasma samples used for the spatial comparison were collected in different years. Two sites, CC and SS, were sampled in spring 2017, SC in spring 2014 and SB-P and CR in spring 2019. For sites with multiple years, data were averaged for the spatial comparisons. In October 2021, SMB were collected at SC and LNC for the tissue distribution analyses.

All fish at a particular site were collected in a single day. Fish were held in aerated live wells until processed (less than 2 h) and euthanized in 350 mg/L Finquel (MS-222, tricaine methanosulfate, Argent Labs, Redmond, WA) following procedures approved by the U.S. Geological Survey Eastern Ecological Science Center’s Institutional Animal Care and Use Committee. Total weight (g), total length (mm), and visible abnormalities were documented, and a blood sample was obtained from caudal vessels using a sterile 3 ml syringe with a 23-gauge needle. Blood was placed into a heparinized Vacutainer tube (Fisher Scientific, Waltham, MA) and stored on wet ice until returned to the laboratory (2–4 h). Blood was centrifuged at 3000 rpm at 4 °C for 10 min and plasma (0.5–1.0 ml) was aliquoted into cryovials and stored at − 80 °C. At the two sites (SC, LNC) for tissue comparison, 1 ml of whole blood and pieces of liver, muscle, and gonad (1–10 gm) were stored on ice in glass vials until returned to the laboratory (2–4 h) and then stored at − 80 °C. Otoliths were removed for age analyses. Adult (mature) fish were collected prespawn, so sex could generally be determined visually; however, gonads were collected for histopathology, and if necessary, sex was determined microscopically (detailed necropsy methodology presented in Blazer et al. (2018)).

The plasma samples collected 2014–2019 were not originally targeted for PFAS analyses. To determine if our methods may have been subject to PFAS contamination, we used PFAS-free water, pulled it into the heparinized syringes, placed into Vacutainers and held on ice for four hours, centrifuged, placed into cryovials, and stored at − 80 °C for 1 week to replicate methodology used to obtain the plasma. PFAS were nondetectable; verifying these methods were appropriate for collection and down-stream PFAS analyses.

### Chemical analyses

Plasma samples collected from 2014 to 2019 were shipped on dry ice to SGS AXYS Analytical Services Ltd., Sidney, British Columbia, Canada. Thirteen analytes (shaded compounds in Table 1) were measured by the SGS AXYS Method MLA-042: analytical procedure for the analysis of perfluoroalkyl carboxylates and sulfonates and perfluorooctane sulfonamide in blood serum by LC–MS/MS. Samples were spiked with isotopically labeled surrogate standards, cleaned up on SPE cartridges, and analyzed by liquid chromatography/tandem mass spectrometry (HPLC–MS/MS). Final sample concentrations were determined by isotope dilution/internal standard quantification against matrix matched calibration standards carried through the analysis procedure alongside the samples. Results were reported directly as ng/ml and detection limits were in the 0.5–1 ng/ml range for a 0.5-mL plasma sample.

Canada changed regulations regarding importation of fish and fish tissues which could no longer be sent to British Columbia. Consequently, plasma (0.5–1.0 ml), whole blood (1.0 ml), and other tissue samples (3–10 g) collected at SC and LNC were analyzed for a total of 28 compounds (Table 1) by RTI Laboratories, Livonia, Michigan, using DOD/DoE QSM 5.2 and 5.3 and USEPA 1633 draft method. Analyses used LC–MS/MS (liquid chromatography mass spectroscopy) and included method blanks, duplicates, post-digestion spikes, serial dilutions, and all method quality controls. Results were reported in ng/kg wet weight and converted to ng/g. Concentrations below the reporting limit but above the detection limit (ranged from 0.6 to 4.5 ng/g) were used in the data analyses. For spatial comparisons, plasma concentrations in ng/g were converted using the following formula: concentrations in ng/g X 1.06 = concentration in ng/ml.

### Land cover and land use attributes

The upstream catchment of all sites was delineated using the medium resolution National Hydrography Dataset (Version 2.1, EPA and USGS, 2012). Land cover and land use (Table 2) were calculated using the 2019 release of the National Land Cover Database (https://www.mrlc.gov/data; Homer et al. 2020). Total low estimated agricultural pesticide application (in kg) was calculated per catchment by year by dividing total applications per compound to appropriate crop types as defined by Wieben (2021). Data on biosolids, defined as treated waste from wastewater treatment plants that are applied to agricultural land, were available from the Chesapeake Scenario Assessment Tool (CAST; Chesapeake Bay Program 2020). Data are estimated as nutrient (nitrogen and phosphorus) applications from biosolids at the county scale for all counties intersecting the Chesapeake Bay watershed for the progress years 2017–2021. Progress year is defined as July 1–June 30; for instance, 2019 data are from July 1, 2018, to June 30, 2019. The application rate (pounds/acre) was applied to the square meters (converted to acres) of pasture, turf grass landcovers (tree canopy over turf grass, large fractional turf grass), and cropland/pasture per catchment and is reported in kg.

**Table 2:** Land use characteristics of the upstream watershed of the sites for spatial comparison

Characteristics	CR^1^	SB-P	SB-M	AC	WBMC	PC	CC	SC	LNC
*Drainage area (sq. km)*	2228.0	2207.7	3150.6	755.8	218.4	2436.8	289.5	1164.8	71.3
*NLCD land cover (2019)*									
Developed	4.5	3.7	3.2	17.3	6.8	3.5	6.9	13.9	70.7
Agricultural^2^	2.8	15.8	14.0	49.1	31.6	8.5	59.4	39.8	9.4
*Pasture/hay*	2.8	15.3	12.6	21.0	12.0	7.7	17.2	14.6	8.2
*Cultivated crops*	0.0	0.5	1.4	28.1	19.6	0.8	42.2	25.2	1.2
Forested	84.8	78.7	80.6	32.2	60.1	84.3	30.5	42.2	15.9
Other^3^	7.9	1.8	2.2	1.4	1.5	3.7	2.1	3.4	4.0
Total PFAS facilities^4^	4	1	5	25	2	23	0	39	60
Biosolid application^5^	ND^7^	2	2	20,241	804	1969	1454	146,596	ND
Pesticide application^6^	2742	13,431	26,815	62,270	11,218	8835	24,621	72,730	375

^1^*CR* Cheat River, *SB-P* South Branch at Petersburg, *SB-M* South Branch at Moorefield, *AC* Antietam Creek, *WBMC* West Brach Mahantango Creek, *PC* Pine Creek, *CC* Chillisquaque Creek, *SC* Swatara Creek, *LNC* Little Neshaminy Creek

^2^Total of pasture/hay and cultivated crops

^3^Other includes woody and emergent wetlands, shrub/scrub, grassland/herbaceous, water, and barren

^4^PFAS transfers, TRI releases, response locations, industrial facilities, federal sites, and discharging facilities

^5^Estimated nutrient (nitrogen and phosphorus) applications from biosolids in kg/year for 2019

^6^Estimated total pesticide application in kg/year for 2019

^7^ND no data available

Locations with known or suspected PFAS storage or releases were downloaded from the EPA PFAS Analytic Tool on 3/21/2023 (https://echo.epa.gov/trends/pfas-tools#data). Points were displayed in ArcPro and joined to the upstream watersheds delineated for each site. The upstream number of PFAS transfers, TRI releases, response locations, industrial facilities, federal sites, and discharging facilities were summed as PFAS sources (Gordon 2024).

### Statistical analyses

Statistical analyses were run using GraphPad Prism version 10.0.2 (GraphPad Software, Boston, MA). One-way ANOVA followed by the Kruskal–Wallis multiple comparison test was used to compared endpoints among multiple sites. Comparison between sexes at each site were made using the nonparametric Mann–Whitney test. Statistical analyses were only conducted on those compounds detected in 100% of the samples and total PFAS which was the sum of all compounds above the detection limit. For all statistical analyses, results were considered significant with a *p* value ≤ 0.05.

## Results

### Spatial comparison: plasma

**Fig. 2:**
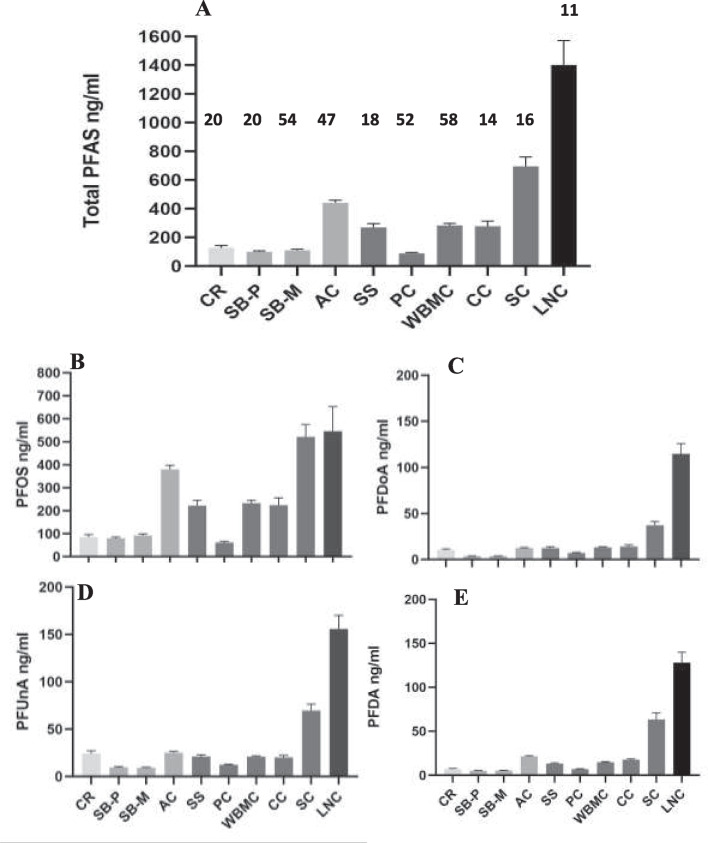
Spatial distribution of PFAS in smallmouth bass plasma. **A** Total PFAS, **B** PFOS, **C** PFDoA, **D** PFUnA, and **E** PFDA, at sites in the Cheat River (CR); South Branch Petersburg (SB-P), South Branch Moorefield (SB-M), and Antietam Creek (AC) in the Potomac River; Susquehanna River Selinsgrove (SS), Pine (PC), West Branch Mahantango Creek (WBMC), Chillisquaque Creek (CC), and Swatara Creek (SC) in the Susquehanna River; and Little Neshaminy Creek (LNC) in the Delaware River. Numbers indicate the sample size of each site

The complete set of age, sex, morphometric, and PFAS concentrations for all fish is available at Blazer et al. (2024). Four compounds (PFOS, PFDA, PFUnA, PFDoA; see Table 1 for definitions) were detected in every plasma sample. PFOS was detected at the highest concentration in all plasma samples ranging from a low of 61.9 ± 5.3 ng/ml (mean ± SEM) at PC to a high of 546.4 ± 106.4 ng/ml at LNC and contributed from 52 to 91% of the total PFAS (sum of detected analytes) concentrations. Three sites, AC, SC, and LNC had the highest concentrations of PFOS (means greater than 300 ng/ml) and total PFAS (means greater than 400 ng/ml). Total PFAS reached concentrations of 1401.0 ± 170.7 ng/ml at LNC. Three other sites, SS, WBMC, and CC, had moderate concentrations of PFOS and total PFAS (means between 200 and 300 ng/ml), while four sites (CR, SB-P, SB-M, and PC) had mean concentrations of PFOS below 100 ng/ml and total PFAS below 150 ng/ml (Fig. 2 A and B).

The three other compounds consistently detected were at considerably lower concentrations than PFOS, often an order of magnitude lower. For these compounds, SC and LNC had the highest, and SB-P and SB-M had the lowest concentrations. PFDA ranged from a mean of 5.1 ± 0.3 ng/ml at SB-P to 128.0 ± 12.0 ng/ml at LNC. PFDoA ranged from a mean of 3.7 ± 0.3 ng/ml at both SB sites to 115.0 ± 11 ng/ml at LNC, while PFUnA mean concentrations ranged from 9.0 ± 0.6 ng/ml at SB-M to 155.7 ± 14.3 ng/ml at LNC. CR and AC had similar moderate concentrations of PFUnA and PFDoA, while PFDA concentrations were low in SMB from CR (Fig. 2 C, D, and E).

Four other compounds, PFNA, PFOSA, PFBA, and PFPeA, were detected less frequently and at lower concentrations. In all cases, concentrations for these four compounds were less than 5 ng/ml. PFNA was detected in 5.5% of the samples at SB-M, 5.0% at SB-P, 27.9% at AC, 13.6% at WBMC, 21.9% at PC, 42.8% at CC, 93.8% at SC, 5.6% at SS, 5.0% at CR, and 0% at LNC. PFOSA was not detected at SB-M, SB-P, SC, or LNC but was detected in 73.8% at AC, 20.3% at PC, 66.7% at SS, 93.9% at WBMC, and 100% at CC. PFBA was only detected in 3.3% at AC and 9.1% at LNC, and PFPeA only detected in 1.6% of the samples at AC.

### Land cover and land use attributes

Land use varied among the sites, as did potential upstream PFAS sources (Table 2). Developed land cover was highest in the upstream catchment of LNC (70.7%), followed by AC (17.3%) and SC (13.9%). These three sites also had the highest number of potential PFAS sources with 60 in LNC, 25 in AC, and 39 in SC catchments. The sites with moderate concentrations of total PFAS and PFOS (WBMC, CC) were smaller watersheds (218.4 to 289.5 sq. km) that had few PFAS facilities (0–2) and low percentage (6.8 to 6.9%) of developed land cover. They did have high agricultural land use (59.4 and 31.6%) with high percentages of cultivated crops, as well as high pesticide and biosolid application rates. The four sites (CR, PC, SB-P, SB-M) with the lowest concentrations of PFOS and total PFAS had the highest drainage areas (3150.6 to 2207.7 sq km), the lowest developed land cover (3.2–4.5%), moderate to low agricultural land cover, and low number of PFAS facilities (1–5), except for PC which had 23 facilities. In the PC watershed, 16 of the 23 facilities were oil and gas operations and three inactive waste management facilities (Table 2).

### Temporal comparisons: plasma

Data from all years were combined to assess PFAS concentrations in association to fish length, sex, and age at the four temporal sites (SB-M, AC, WBMC, PC). A comparison of male and female at each site indicated that neither age nor total length was significantly different between the sexes at any site. There were also no site differences in the age of either sex; however, mean length of both sexes from WBMC were significantly greater than those collected at the other three sites (Table 3).

**Table 3:** Age, length, sex, and site comparisons of smallmouth bass collected at the four temporal sites^1^

Site	Age		Length	
	Female	Male	Female	Male
Antietam Creek	3.9 ± 0.2 (24)	3.8 ± 0.2 (23)	308.3 ± 8.9^a^	298.4 ± 7.9^a^
South Branch Moorefield	4.4 ± 0.3 (33)	4.2 ± 0.3 (21)	315.3 ± 9.9^a^	323.6 ± 12.1^a^
West Branch Mahantango	4.6 ± 0.3 (24)	4.5 ± 0.3 (34)	372.2 ± 8.1^b^	360.8 ± 5.8^b^
Pine Creek	4.8 ± 0.3 (!5)	4.1 ± 0.3 (37)	297.8 ± 14.9^a^	317.3 ± 8.5^a^

^1^Data presented as mean ± standard error. Values followed by the same letter are not significantly different (*p* < 0.05). Numbers in parentheses are the sample sizes

Male SMB consistently had higher mean concentrations of all compounds than females, except PFOS at AC, but concentrations were not always significantly higher and the relationship varied by compound and site (Table 4). There was no significant difference between the sexes at any site for PFDA concentrations. PFOS concentrations were not significantly different between males and females at PC and SB-M (sites with the lowest concentrations), while there were sex difference at WBMC and AC sites. Males had higher concentrations at WBMC, while at AC females had higher concentrations. Male plasma concentrations of PFUnA and PFDoA were significantly higher than female concentrations at all sites (Table 4).

**Table 4:** Site and sex comparisons for plasma concentrations of PFOS, PFDA, PFUnA, and PFDoA collected at the four temporal sites

Site	Female	Male	Sex difference^1^
PFOS			
Pine Creek	50.1 ± 4.6^a^ (15)	66.7 ± 7.1^a^ (37)	ns
WB Mahantango Creek	194.3 ± 12.9^b^ (24)	261.2 ± 19.3^b^ (34)	*p* = 0.0210
Antietam Creek	414.8 ± 25.8^c^ (24)	344.6 ± 23.0^b^ (23)	*p* = 0.0278
South Branch Potomac	88.8 ± 8.2^a^ (33)	97.6 ± 10.9^a^ (21)	ns
PFDA			
Pine Creek	6.4 ± 0.9^a^	7.0 ± 0.6^a^	ns
WB Mahantango Creek	13.4 ± 0.7^b^	15.8 ± 1.0^b^	ns
Antietam Creek	22.2 ± 1.3^b^	20.4 ± 1.4^b^	ns
South Branch Potomac	5.2 ± 0.3^a^	5.7 ± 0.5^a^	ns
PFUnA			
Pine Creek	8.7 ± 0.8^a^	13.6 ± 0.7^a^	*p* < 0.0001
WB Mahantango Creek	15.7 ± 0.6^b^	24.3 ± 1.4^b^	*p* < 0.0001
Antietam Creek	21.7 ± 1.0^b^	28.6 ± 2.4^b^	*p* = 0.0329
South Branch Potomac	7.5 ± 0.6^a^	11.5 ± 0.9^a^	*p* < 0.0001
PFDoA			
Pine Creek	4.8 ± 0.7^a^	8.1 ± 0.6^a^	*p* = 0.0006
WB Mahantango Creek	9.6 ± 0.5^b^	15.5 ± 0.9^b^	*p* < 0.0001
Antietam Creek	10.2 ± 0.5^b^	14.8 ± 1.3^b^	*p* = 0.0022
South Branch Potomac	2.8 ± 0.2^a^	5.0 ± 0.4^a^	*p* < 0.0001

^a^Values followed by the same letter indicate sites are not significantly different at *p* < 0.05. Numbers in parentheses indicate sample size which was the same for all compounds

^1^A difference between female and male at each site. *ns* no significant difference

**Fig. 3:**
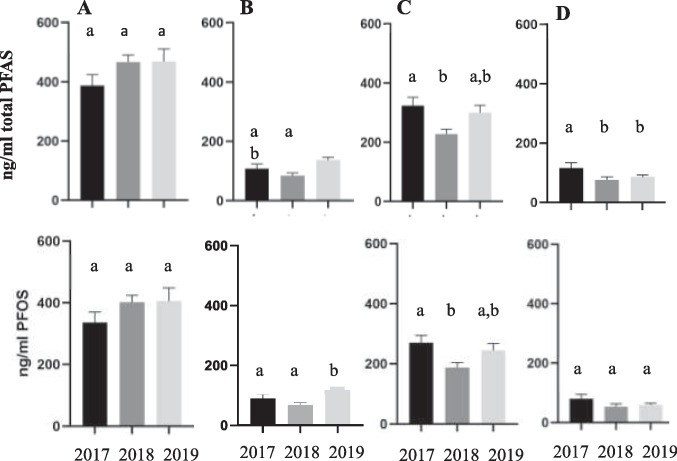
Annual difference in total PFAS (top panel) and PFOS (bottom panel) in Smallmouth Bass plasma concentrations (ng/ml) collected at **A** Antietam Creek (AC); **B** South Branch Potomac, Moorefield (SB-M); **C** West Branch Mahantango Creek (WBMC); and **D** Pine Creek (PC). Presented as mean with standard error bars. Years with the same letter are not significantly different at *p* < 0.05

Concentrations of certain plasma PFAS varied over time at all sites and overall patterns of PFOS and total PFAS were similar. Although concentrations of both increased over time at AC, there were no significant differences among the years (Fig. 3A). Concentrations of both were significantly higher at SB-P in 2019 than 2017 and 2018 (Fig. 3B). Concentrations of both at WBMC were significantly higher in 2017 than 2018, with 2019 was intermediate (Fig. 3C). At PC, total PFAS concentrations were higher in 2017 than 2018 and 2019, but PFOS concentrations were not significantly different among years (Fig. 3D).

**Fig. 4:**
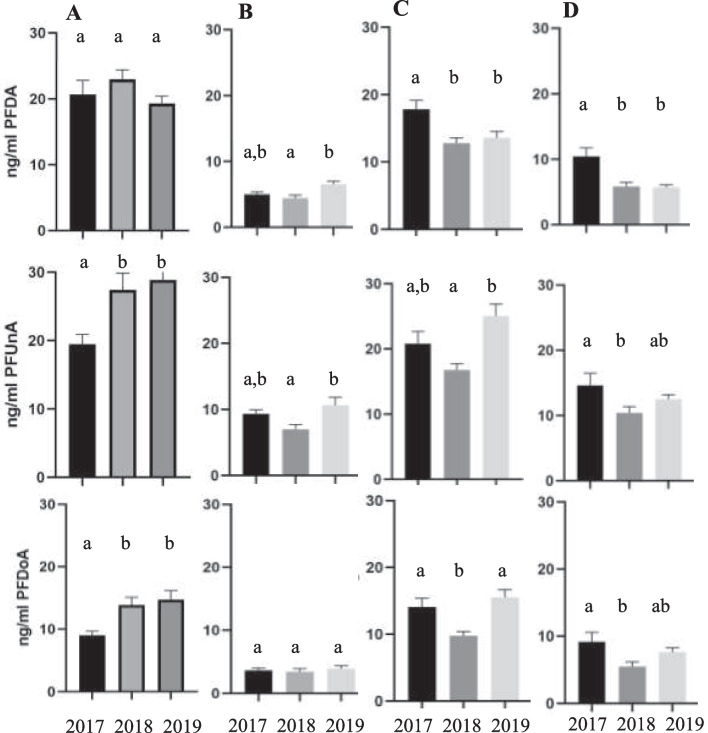
Annual differences in PFDA (upper panel), PFUnA (middle panel), and PFDoA (bottom panel) plasma concentrations in Smallmouth Bass from **A** Antietam Creek (AC); **B** South Branch Potomac at Moorefield (SB-M); **C** West Branch Mahantango Creek (WBMC); and **D** Pine Creek (PC). Data (in ng/ml) presented as means with standard error bars. Bars with the same letter are not significantly different at *p* < 0.05

The other three PFAS, while at lower concentrations, also showed variation over time, but did not always show the same patterns. At AC, concentrations of PFDA did not differ among years, while PFUnA and PFDoA were significantly lower in 2017 than 2018 or 2019 (Fig. 4A). At SB-M, concentrations of PFDA and PFUnA were highest in 2019, lowest in 2018, and intermediate in 2017, while there was no difference among years for PFDoA (Fig. 4B). At WBMC, concentrations of PFDA were higher in 2017 than the other 2 years, while concentrations of PFUnA and PFDoA were lowest in 2018 (Fig. 4C). At PC, the highest concentrations for all three compounds were observed in 2017 (Fig. 4D).

Pesticide and biosolid applications were compared among years to better understand potential sources. Pesticide application in the upstream catchments was highest in 2018 for all sites, except SB-M, which was highest in 2019. Biosolid application was low in the upstream catchment of the SB-M and did not change over time. Biosolid application was highest at AC and increased over time. It increased slightly over time at PC but was similar among the years at WBMC (Table 5).

**Table 5:** Changes in certain land use practices in the upstream catchment of the four temporal sites

	Antietam Creek	South Branch Moorefield	West Branch Mahantango Creek	Pine Creek
Pesticide application^1^				
2017	79,183	24,788	11,698	6640
2018	81,997	23,887	12,048	9139
2019	62,270	26,815	11,218	8835
Biosolid application^2^				
2017	18,825	2	803	1949
2018	18,834	2	803	1959
2019	20,241	2	804	1969

^1^Total pesticide application in kg/year

^2^Estimated nutrient (nitrogen and phosphorus) application for biosolids in kg/year

### Tissue distribution of PFAS

Distribution of PFAS in various tissues including the plasma, whole blood, liver, gonad, and muscle was compared in 17 adult bass (7 female and 10 male) collected from SC and 11 adult bass (6 female and 5 male) collected from LNC in Fall 2021. Although sample sizes were low, no significant differences were observed between the sexes when comparing individual tissues; therefore, sexes were combined for further analyses. Eight compounds (PFNA, PFBS, N-MeFOSAA, N-EtFOSAA, 4:2 FTS, ANONA, 9C1-PF3ONS, 11C1-PF3OYUds) were not detected in any tissue at either site. Twelve compounds were detected at least once at SC; nine of these were also detected at LNC. Three compounds (PFHxA, PFOA, 8:2 FTS) were detected only at SC and only rarely (Table 6). Seven compounds were only rarely detected at LNC except PFNS, which was detected in 82% of plasma samples with a lower incidence in other tissues. PFOSA was detected in 73% of whole blood samples and 9% of liver samples at LNC (Table 7).

**Table 6:** Detection frequency (%) and concentration range (ng/g) of PFAS in tissues of 17 smallmouth bass from Swatara Creek, Susquehanna River drainage

Compound	Plasma	Blood	Liver	Gonad	Muscle
PFOS	100 88–520	100 66–210	100 27–130	100 14–82	100 5.4–30
PFDA	100 7.2–38	100 6–17	100 2.1–6.3	82 BD^1^–6.2	24 BD–3.2
PFUnA	100 8.8–53	100 11–25	100 3.3–5.9	100 0.5–9.3	71 BD–2.3
PFDoA	88 BD–23	47 BD–13	47 BD–4.4	0 BD	0 BD
PFDS	94 BD–8.8	24 BD–3.5	18 BD–2.9	18 BD–1.5	0 BD
PFTeDA	94 BD–19	12 BD–8.0	29 BD–2.6	18 BD–2.8	0 BD
6:2 FTS	71 BD–6.6	0 BD	0 BD	88 BD–4.8	0 BD
PFTrDA	35 BD–8.2	41 BD–15	6 BD–1.5	0 BD	0 BD
Gen X	0 BD	18 BD–7.8	29 BD–45	0 BD	6 BD–13
PFHxA	0 BD	0 BD	12 BD–3.8	0 BD	0 BD
PFOA	6 BD–3.7	6 BD–4.5	0 BD	0 BD	0 BD
8:2 FTS	6 BD–1.0	0 BD	0 BD	0 BD	0 BD
*Total* *detections*	*118*	*76*	*75*	*69*	*34*

^1^*BD* below detection

**Table 7:** Detection frequency (%) and concentration range (ng/g) of PFAS in tissues of 11 smallmouth bass from Little Neshaminy Creek, Delaware River drainage

Compound	Plasma	Blood	Liver	Gonad	Muscle
PFOS	100 250–1200	100 680–4000	100 500–6000	100 230–1100	100 81–520
PFDA	100 86–200	100 49–84	100 11–29	100 10–21	100 3.1–9.3
PFUnA	100 96–240	100 36–110	100 20–40	100 17–32	100 4.6–13
PFDoA	100 67–160	91 BD^1^–76	100 12–27	100 9.7–27	18 BD–4.6
PFDS	100 12–82	91 BD–35	100 8.1–25	100 4.3–14	45 BD–9.3
PFTeDA	91 BD–70	91 BD–37	100 8.5–18	91 BD–16	64 BD–7.2
6:2 FTS	100 3.6–1300	100 26––130	100 18–29	0 BD	0 BD
Gen X	18 BD–6.7	9 BD–12	0 BD	0 BD	9 BD–6
PFHpS	36 BD–1.5	36 BD––3.6	0 BD	0 BD	0 BD
PFHxS	18 BD–1.5	0 BD	0 BD	0 BD	0 BD
PFNS	82 BD–22	45 BD–12	36 BD–4.7	27 BD–2.7	9 BD–2.6
PFOSA	0 BD	73 BD–37	9 BD–3.6	0 BD	0 BD
PFPeS	0 BD	0 BD	0 BD	18 BD–2.9	0 BD
PFPeA	0 BD	0 BD	0 BD	0 BD	0 BD
PFBA	9 BD–1.5	0 BD	9 BD–1.6	0 BD	0 BD
*Total detections*	*101*	*102*	*93*	*78*	*49*

^1^*BD* below detection

PFOS was detected in all tissues at both sites. PFDA and PFUnA were detected in every plasma, whole blood and liver sample at both sites and in all gonad and muscle samples at LNC. At SC, PFDuA was detected in all gonad samples. PFDoA, PFDS, and PFTeDA were detected at a high frequency (88–94%) in plasma; a lower frequency in whole blood, liver, and gonad; and not detected in muscle at SC. At LNC, these three compounds were detected in 91–100% of plasma, blood, liver, and gonad with less frequency in muscle. PFTrDA was detected in 91–100% of the whole blood and liver, 64% in plasma and gonad, and 0% of muscle samples at LNC. One compound, 6:2 FTS was detected in all plasma, whole blood, and liver tissues from LNC but was not detected in gonad or muscle. At SC, the highest frequency of detection (88%) of 6:2 FTS was in the gonad, followed by plasma (71%) with no detections in other tissues. At SC, plasma had the highest frequency of 6:2 FTS detections followed by whole blood, liver, and gonad, while muscle had the lowest (Table 6). At LNC, detections were highest in whole blood and plasma, followed by liver and gonad and lowest in muscle (Table 7).

**Fig. 5:**
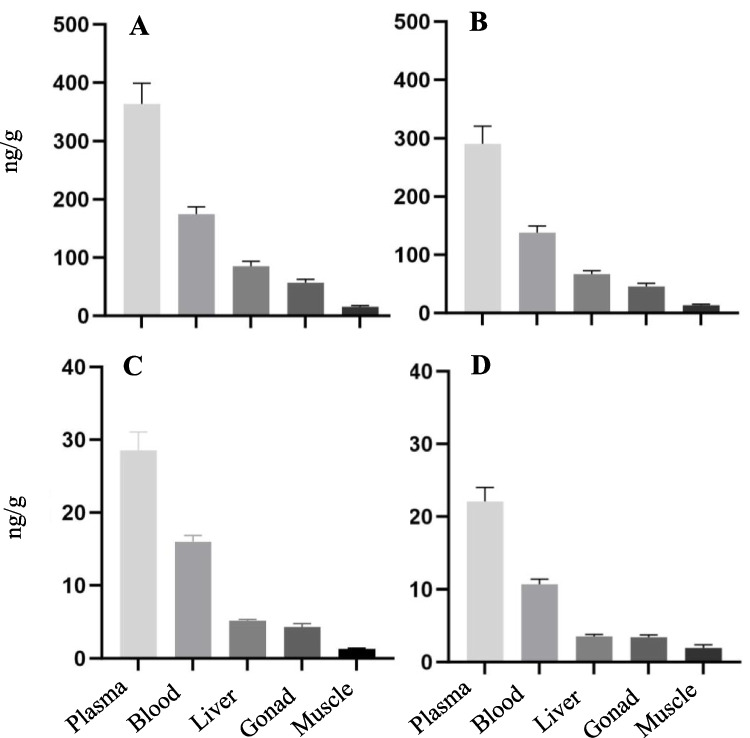
Tissue concentrations (mean in ng/g with standard error bars) in 17 smallmouth bass collected from Swatara Creek (SC), Pennsylvania. **A** Total PFAS; **B** PFOS; **C** PFUnA; **D** PFDA

**Fig. 6:**
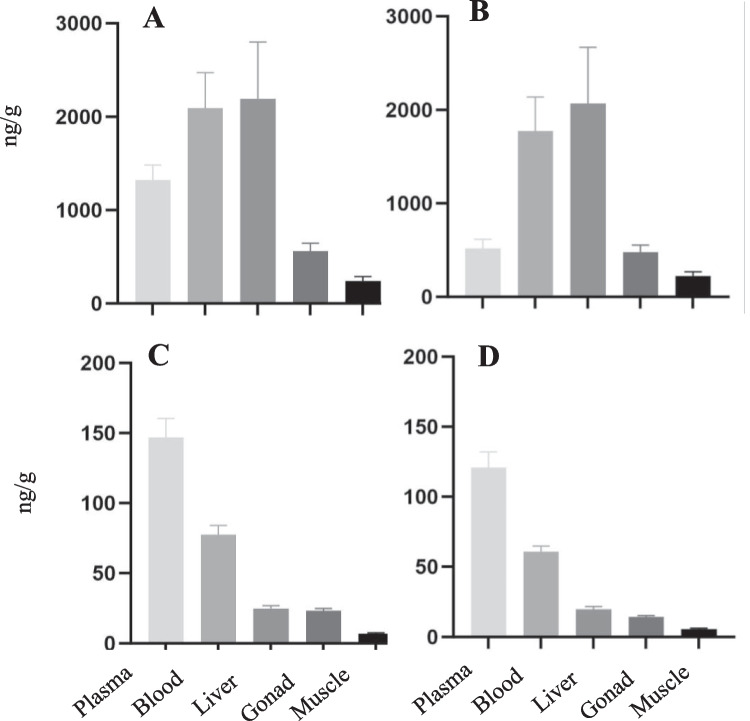
Tissue distribution of specific PFAS compounds (in ng/g mean with standard error bars) detected in 11 smallmouth bass from Little Neshaminy Creek (LNC), Pennsylvania. **A** Total PFAS. **B** PFOS. **C** PFUnA. **D** PFDA

Total PFAS concentrations at SC were highest in plasma, followed by whole blood, liver, gonad, and muscle (Fig. 5A), as were PFOS (Fig. 5B), PFUnA (Fig. 5C), and PFDA (Fig. 5D). For compounds not detected in 100% of muscle and gonad samples, mean concentrations were based only on the samples above detection limits. If non-detects in muscle and gonad had been estimated either as zero or a percentage of the detection limit, these values would have been even lower. These three compounds were also detected in all tissues from LNC, but at significantly higher concentrations than measured at SC. The pattern of tissue distribution for PFUnA (Fig. 6C) and PFDA (Fig. 6D) was the same between the two sites; however, the pattern for PFOS (Fig. 6B) and, consequently, total PFAS (Fig. 6A) were different. At LNC mean liver concentrations were highest, followed by whole blood, plasma, gonad, and muscle, respectively.

## Discussion

The plasma concentrations of PFAS in SMB varied considerably, across sites and by compound. Concentrations of PFAS can increase at higher trophic levels due to biomagnification (Martin et al. 2004; Miranda et al. 2022; Munoz et al. 2022), and SMB, as an apex predator, has been found in other studies to have high concentrations of PFOS and total PFAS, relative to other species. They had the highest concentrations (in fillets) of four species tested in Delaware (MacGillivray 2021) and of 14 species tested (whole body) in the St. Lawrence River (Munoz et al. 2022). However, due to the differences in tissue analyzed (fillets, whole body, plasma), it is difficult to compare concentrations across studies. Additionally, some studies include estimates for non-detections (or below detection) values, and a number of approaches have been used to include these values in statistical comparisons. Some researchers have commonly substituted one-half the detection or quantitation limit for non-detections, while other studies use more detailed computational methods. The approach can influence conclusions (Helsel 2006; Miranda et al. 2022) and influence comparability among studies. In this study, we focused on those compounds detected at a high frequency for comparisons of concentrations among sites and tissues and did not estimate a value for non-detections. Statistical comparisons were only made for the four compounds (PFOS, PFDoA, PFUnA, PFDA) with 100% frequency.

The results of the current research indicated that SMB plasma and blood are appropriate, nonlethal media to evaluate exposure and uptake of both legacy and emerging PFAS. Compounds were most commonly detected and often at the highest concentrations in plasma and whole blood. Fewer compounds were detected in muscle (fillet) and at much lower concentrations (Tables 6 and 7). Fish consumption is one of the main routes of PFAS (particularly PFOS) exposure for humans (Domingo and Nadal 2017; Barbo et al. 2023). Therefore, fillets are often the tissue of choice for analyses. Although muscle (fillet) is important in terms of human health concerns and biomagnification in fish-eating organisms, our results indicate that muscle concentrations may not be sufficient for assessing potential effects on fish populations. Understanding the internal exposure to various tissues and cells is necessary for identifying potential adverse effects. Sportfish, such as SMB, may be a human food source for subsistence fishers and others (Liu et al. 2022), but are also economically important in many states (Brewer and Orth 2015; Schall et al. 2020; Keplinger and Rota 2024). Therefore, understanding bioaccumulation, tissue distribution, and potential adverse effects of PFAS, independently and in combination with other stressors is important.

Four of the 13 compounds analyzed in plasma, PFOS, PFDA, PFUnA, and PFDoA, were detected in all samples during the spatial assessment. Reports on PFAS most commonly detected in fish vary; and detections are most likely due to differences in exposure sources (chemical composition), species and tissues analyzed. PFOS has previously been reported as the most commonly detected in fish tissue worldwide (Houde et al. 2006; Lin et al. 2021). Whole body PFAS concentrations were measured in 14 fish species in the St. Lawrence River food web. PFOS accounted for approximately 81% of the total, with PFDA, PFUnA, and PFDoA the other major compounds detected (Munoz et al. 2022). These four compounds were also the most detected in fillets of multiple fish species from New Jersey waterbodies (Goodrow et al. 2020). In general, in Great Lakes fishes, PFUnA was the second most abundant compound detected, followed by PFDA and PFNA (Lin et al. 2021).

PFOS was also detected at the highest concentrations in the current study. Individual PFOS concentrations in plasma ranged from a low of 19.7 ng/ml at PC to a high of 1272 ng/ml at LNC. While PFOS is directly produced during manufacturing, it is also the terminal/metabolized product for numerous parent or precursor compounds. The persistent, longer-chained perfluoroalkyl-carboxylic acids (PFDA, PFDoA, and PFUnA) and sulfonic acids (PFOS) are considered persistent and not capable of biotransformation (i.e., terminal). However, numerous parent or precursor compounds, such as PFOSA and N-EtFOSA, can be metabolized to PFOS. Emerging precursors such as PFBS, PFOSA, N-EtFOSA, and N-MeFOSAA were found in numerous fish species collected in New Hampshire, and it was suggested they may be bioaccumulative (Pickard et al. 2022). PFBS was not detected in SMB plasma samples but the analyte list for most of the plasma samples did not include N-EtFOSA and N-MeFOSAA. They were also not detected with the expanded analyte list used for tissues. PFOSA was detected in plasma at a relatively high frequency at certain sites such as CC (100%), WBMC (94%), and AC (74%) during the spatial analyses, although in all cases concentrations were below 5 ng/ml. PFOSA can be metabolized to PFOS, and it has been suggested that the presence of PFOSA in tissue may indicate a limited capacity for biotransformation in certain fish species (Houde et al. 2006; Babut et al. 2017). Lin et al. (2021) noted higher concentrations of PFOSA in walleye (*Sander vitreus*), than SMB collected at the same sites, suggesting the SMB more readily metabolize PFOSA. It is currently unknown whether the site differences observed in the current study indicate a more recent exposure at sites with a high frequency of detection, site differences in exposure (water/sediment versus diet), specific sources, or metabolic differences among the sites. It should be noted that these three sites also had high percentages of agricultural land use in the upstream catchments. PFOSA has been reported in biosolids (Venkhatesan and Halden 2014) and is a degradation/metabolite product of N-EtFOSAA, the active ingredient of the insecticide sulfluramid (Nascimento et al. 2018).

Fish and other aquatic organisms can uptake PFAS from water (Martin et al. 2003a; Shi et al. 2018), sediment (Munoz et al. 2017), and diet (Martin et al. 2003b; Goeritz et al. 2013; Gaillard et al. 2017). Some studies have suggested a correlation between long-chain PFAS and fish length and/or age (Gewurtz et al. 2014; Pan et al. 2014), while others have not observed these relationships (Ye et al. 2008; Fair et al. 2019). In the current study, no correlation of long-chained PFAS with age or length was observed at the four temporal sites; however, these were all mature (length > 200 mm and visibly developed gonads) adult individuals. Perhaps if juveniles were included, length or age may have been factors. There were, however, sex-related differences. The plasma concentrations of PFUnA and PFDoA were higher in males than females at all sites. There was no difference between the sexes at any site for PFDA, and findings for PFOS varied by site. Brown et al. (2023) also found higher concentrations in male largemouth bass (*Micropterus salmoides*) liver for a number of PFAS when compared to females. Sinclair et al. (2006) reported higher concentrations of PFOS in male SMB when compared to female in remote lakes considered to have background contamination and hypothesized that this may be due to oviparous elimination. In the current study, PFOS was detected in both male and female gonads in the fall (during recrudescence). However, the sites used for sex comparisons were all collected in spring during the pre-spawn period, and only plasma was analyzed. Further analyses of ovaries/eggs will be necessary to determine if sex differences are related to elimination through eggs. Some studies (Martin et al. 2004; Sedlak et al. 2017) found that benthic dwelling fish have higher concentrations than pelagic fishes and hypothesized that PFAS-contaminated sediments are an important source. Higher sediment concentration, relative to water, have been reported (Pignotti et al. 2017). Spring/prespawn is a time when male SMB are in contact with stream sediment while building and guarding nests (Dauwalter and Fisher 2007), which could explain the higher concentrations in males, particularly of the longer chain PFAS which are often sediment-bound. Alternatively, it has been proposed that adult male fishes have higher contaminant concentrations (polychlorinated biphenyls) due to a higher rate of energy expenditure leading to a higher rate of food consumption (Madenjian et al. 2016). Further studies during different seasons and evaluating different tissues will be necessary to better elucidate sex-specific differences in SMB.

Considerable variation observed among sites in the current study appeared to be associated with land cover and potential PFAS sources in the upstream catchment. The three sites with the highest concentrations of total PFAS and PFOS (LNC, SC, AC) also had the highest percentages of developed land and the highest number of PFAS facilities. At LNC and SC, sites with the highest concentrations, these facilities included defense facilities (National Guard Units and Naval Joint Reserve Base). The upstream catchment of AC contained an airport. These facilities can be important point sources of PFAS due to the use of AFFF or aqueous fire-fighting foam (Leeson et al. 2021). SC and AC had lower developed land than LNC, but both had a high percentage of agricultural lands in cultivated crops with high levels of biosolid and pesticide application, both potential sources of PFAS in agricultural settings (reviewed by Ye et al. 2024). The sites with the moderate to low concentrations of PFAS also had high agricultural land use, suggesting that further study is needed on agricultural inputs in this region, in addition to potential atmospheric and groundwater sources. PC, with low PFAS concentrations in SMB plasma, had a high number (23) of potential PFAS sources, most of them unconventional oil and gas facilities that may or may have been active during the sampling period. An assessment of PFAS (33 analytes) in surface waters (161 streams) throughout Pennsylvania in 2019 found that the percentage of development in the upstream catchment was the primary factor explaining total PFAS concentrations. However, agricultural landscape and number of water pollution control facilities were also important explanatory factors (Breitmeyer et al. 2023). Our findings support those of Breitmeyer et al. (2023) and suggest that agriculture may be an important source at sites with less development and fewer point sources.

Temporal analyses at PC, WBMC, AC, and SB-M revealed annual variation, although often not significant and in no consistent pattern. Only 3 years of data were available, and a much longer time period would be necessary to accurately determine temporal changes. However, the relative relationship of the sites to each other over the 3 years was consistent with AC having the highest concentrations, followed by WBMC, with concentrations at PC and SB-M the lowest in all years. The variations did not correlate with total pesticide application or the estimated biosolid application in the upstream catchment. However, these are gross estimates that do not take into account the types of pesticides used or the sources of biosolids. Temporal (seasonal) changes in water have been reported in some studies to be related to environmental conditions such as rainfall and temperature (Pignotti et al. 2017). It seems likely that there are multiple and complex relationships regulating temporal changes in water, sediment, and biota that include the sources and types of PFAS compounds, exposure routes (i.e., runoff or atmospheric versus point sources), habitat, and species of concern. Longer-term monitoring that includes water and sediment could help improve understanding of temporal variation.

Few studies have compared tissue distribution in SMB tissue. Paired liver and muscle samples were compared in SMB collected from both background (no known PFAS sources) and impacted ecosystems in eastern Canada and higher frequencies of detection and concentrations were observed in liver (Kaboré et al. 2022). There have been studies in other fish species regarding tissue distribution. George et al. (2023) collected nonlethal muscle plugs and serum from walleye, yellow perch (*Perca flavescens*), and round goby (*Neogobius melanostomus*). Eight compounds were detected in muscle versus 15 in serum with PFOS having the highest frequency of detection and concentration. Although a number of previous studies have demonstrated higher concentrations in plasma/serum, followed by liver with lower concentrations in the muscle (Shi et al. 2012; Goeritz et al. 2013; Houde et al. 2013; Nilsen et al. 2024), higher concentrations in the liver than blood were reported by Hung et al. (2020), similar to our findings at LNC.

The difference in tissue distribution of PFAS between SC and LNC suggests that factors other than species influence tissue distribution. In aqueous exposure experiments of rainbow trout (*Oncorhynchus mykiss*) to a mixture of PFAS, accumulation was greatest in the blood and then kidney, liver, gonad, and muscle (Martin et al. 2003a). Conversely, rainbow trout exposed through diet contained the highest concentrations of PFOS in liver (Martin 2003b; Falk et al. 2015). Hence, SMB a the LNC site may be obtaining a higher percentage of PFAS through their diet, leading to higher concentrations in the liver.

In rainbow trout, plasma contained over 90% of the PFAS with only a minor fraction detected in the blood cells (Martin 2003b). The difference in PFAS concentrations between whole blood and plasma at SC and LNC is especially interesting. In humans, diet and drinking water are the two primary exposure routes, and hence PFAS are absorbed through the gastrointestinal tract and distributed throughout the body via blood circulation. These compounds preferentially bind to proteins such as serum albumin, with accumulation mainly in the blood (plasma or serum), liver, and kidney (reviewed by Lu et al. 2024). Albumin was determined to be the major carrier for PFOS, PFOA, PFHxS, PFNA, and PFDA (Forsthuber et al. 2020), while PFOSA and PFHxA were higher in whole blood of humans (Poothong et al. 2017). PFOSA was detected in whole blood, but not plasma of SMB at LNC. Further studies on the binding of various PFAS to fish plasma proteins and blood cells and the factors which influence this binding will be necessary to better understand the transport and accumulation of individual PFAS in fish tissues. The differential tissue distribution between the two sites, in the same species, suggests that contaminant sources, habitat usage, available food sources, and other factors influence tissue accumulation.

The expanded analyte list used for tissue allowed us to measure some additional emerging PFAS. Most were not detected or in the case of 8:2 FTS and Gen X were only occasionally detected. GEN-X was detected in 18% of blood samples and 29% of liver samples at Swatara Creek, with concentrations as high as 45 ng/g. The exception to the low frequency of detection of emerging compounds was 6:2 FTS found in 71% of plasma and 88% of gonad samples at SC, however concentrations were below 10 ng/g. At LNC, it was detected in 100% of plasma, whole blood, and liver samples, and concentrations reached 1300 ng/g in plasma. Hoke et al. (2015) suggested that 6:2 FTS is unlikely to bioaccumulate or biomagnify in aquatic systems. However, our findings indicate that further analyses are warranted.

At SC, total gonad PFAS ranged from 20.2 to 98.4 ng/g, while the range at LNC was 287.0 to 1231.0 ng/g. Experimental exposures of fathead minnows showed both PFOS and PFHxS accumulated in gonad (Ankley et al. 2009; Suski et al. 2021). In SMB, PFOS and PFUnA were detected in 100% of the gonads at both sites. PFDA was detected in 100% at LNC and 82% at SC. Our samples sizes of male and female at each site were too small to detect differences between sexes. However, given the association of PFAS with reproductive endocrine disruption (Chambers et al. 2021; Villeneuve et al. 2023) and the endocrine disruption (intersex and plasma vitellogenin in males) documented in SMB in the Chesapeake Bay drainage (Blazer et al. 2007, 2014), it will be important to further explore the levels detected with associated adverse effects.

In conclusion, the results of this study indicate a significant spatial variation in PFAS concentrations in SMB plasma related to both land use and specific point sources. Temporal variation over a 3-year period occurred at four sites, but the patterns varied by site and compound. Our research supports the hypothesis that location (sources, environmental factors) not only influence uptake and transport in the plasma but also accumulation in other tissues. Therefore, evaluating associations between PFAS concentrations and biological indicators of fish health, conducting further field analyses as well as laboratory exposures to complex mixtures, will be needed to fully understand potential effects of PFAS in combination to numerous other environmental stressors.

## Data Availability

Blazer, V.S., Walsh, H.L., and Smith, C.R., 2024, Per- and Polyfluoroalkyl Substances (PFAS) Detected in Smallmouth Bass Collected in Mid-Atlantic Watersheds: U.S. Geological Survey data release, https://doi.org/10.5066/P1P6CPPE
